# Is there a difference in bleeding after left ventricular assist device implant: centrifugal versus axial?

**DOI:** 10.1186/s13019-018-0703-z

**Published:** 2018-02-13

**Authors:** Ann C. Gaffey, Carol W. Chen, Jennifer J. Chung, Jason Han, Christian A. Bermudez, Joyce Wald, Pavan Atluri

**Affiliations:** 1 Division of Cardiovascular Surgery, Department of Surgery, Perelman School of Medicine, University of Pennsylvania, Silverstein 6, 3400 Spruce St, Philadelphia, PA 19104 USA; 2 Division of Cardiology, Department of Medicine, Perelman School of Medicine, University of Pennsylvania, Philadelphia, USA

**Keywords:** Left ventricular assist device, Gastrointestinal bleeding

## Abstract

**Background:**

Continuous-flow left ventricular assist devices (CF-LVAD) have become the standard of care for patients with end stage heart failure. Device reliability has increased, bringing the potential for VAD, compared to transplant, into debate. However, complications continue to limit VADs as first line therapy. Bleeding is a major morbidity. A debate exists as to the difference in bleeding profile between the major centrifugal and axial flow devices. We hypothesized that there would be similar adverse bleeding event profiles between the 2 major CF-LVADs.

**Methods:**

We retrospectively investigated isolated CF LVADs performed at our institution between July 2010 and July 2015: HeartMateII (HMII, *n* = 105) and HeartWare (HVAD, *n* = 34). We reviewed demographic, perioperative and short- and long-term outcomes.

**Results:**

There was no significant difference in demographics or comorbidities. There was a low incidence of gastrointestinal (GI) bleed 3.9% in HMII and 2.9% in HVAD (*p* = 0.78). Preoperatively, the cohorts did not differ in coagulation measures (*p* = 0.95). Within the post-operative period, there was no difference in product transfusion: red blood cells (*p* = 0.10), fresh frozen plasma (*p* = 0.19), and platelets (*p* = 0.89). Post-operatively, a higher but not significantly different number of HMII patients returned to the operating room for bleeding (*n* = 27) compared to HVAD (*n* = 6, *p* = 0.35). There was no difference in rates of stroke (*p* = 0.65), re-intubation (*p* = 0.60), driveline infection (*p* = 0.05), and GI bleeding (*p* = 0.31). The patients had equivalent ICU LOS (*p* = 0.86) and index hospitalization LOS (*p* = 0.59).

**Conclusion:**

We found no difference in the rate of bleeding complications between the current commercially available axial and centrifugal flow devices.

## Background

During the past several decades, left ventricular assist devices (LVADs) have become a valuable and indispensable therapeutic option in the management of end stage heart failure. Recent data suggest that one of the most common adverse events within the first 30 days after LVAD implantation is bleeding in particular bleeding not requiring a return to the operating room [1–5]. Data from the Interagency Registry for Mechanically Assisted Circulatory Support (INTERMACS) have shown that the most frequent location of the first bleeding episode after implantation to be mediastinal (45%), thoracic-pleural space (12%), lower gastrointestinal (GI) tract (10%), chest wall (8%), and upper GI tract (8%) with no difference in the overall bleeding rates between axial- and pulsatile flow devices [6].

The etiology of the greater rate of non-surgical bleeding is due to the relatively high non-physiological stress imparted on the blood components as they move through the device. Stress coupled with the reduced pulse pressure may result in the unanticipated increases in nonsurgical bleeding related to arteriovenous malformations (AVMs) in the GI tract. Bleeding specifically from the GI tract has been identified as the most common adverse event in implantation and is a major cause of morbidity in patients supported with LVAD therapy [7–10]. One of the proposed mechanisms for GI bleeding following implantation of an LVAD is acquired von Willebrand syndrome. Von Willebrand factor (vWF) is a protein expressed by vascular endothelial cells that is essential in preventing coagulopathy or bleeding; however, excessive cleavage of large vWF results in bleeding syndrome in the acquired syndrome as seen in patients with LVADs [11]. This paper aims to compare outcomes between axial (HeartMate II) and centrifugal (HeartWare) flow LVADs with respect to bleeding outcomes at a single institution.

## Methods

### Study design

All patients who underwent implantation of a CF-LVAD from July 2010 to July 2015 (*n* = 139) at the University of Pennsylvania were retrospectively reviewed. After initial data accrual, all patient identifiers were removed from the database. The University of Pennsylvania Institutional Review Board (IRB 7) approved this study for investigation.

Device selection was based on patient characteristics and surgeons’ preference. Patients were initially not anti-coagulated on post-operative day zero. On post-operative day one, the patients were started on a heparin drip with a goal partial thromboplastin time of 45–50 s. Ultimately, all patients were anticoagulated with 325 mg of aspirin and Coumadin to a target International Normalized Ratio (INR) of 2.0–3.0. No additional anti-platelet agents were used in either cohort.

Patient demographics, co-morbidities, laboratory values, and complications were retrospectively analyzed using our institutional database. Baseline information was collected the day before LVAD implantation. Patient demographic information and co-morbidities included age, sex, diabetes, etiology of cardiomyopathy, indication for LVAD, and INTERMACS patient profile risk score. Height and weight were collected in order to calculate body mass index (BMI). We also collected cardiopulmonary bypass time, days of mechanical ventilation, duration of hospital stay, and ICU length of stay.

Patients were followed on LVAD support for complications and intraoperative outcomes out to 36 months. Potential device related complications included: GI bleeding, infection, right ventricular failure, stroke, VAD malfunction, and wound infection. We defined GI bleeding by hematemesis, melena, or active bleeding at the time of endoscopy or colonoscopy. All complications were recorded during ICU stay except for device malfunction, GI bleeding, and death, which were also followed after discharge.

### Outcomes analysis

The primary outcomes were post-operative bleeding events. Secondary outcomes included postoperative complications and length of stay outcomes. Normally distributed variables were presented as mean value ± standard deviation; non-normally distributed were presented as median value with interquartile range. Normality of all data variables was tested. Non-parametric data was analyzed using Kruskal-Wallis one way analysis of variance and parametric data was analyzed by a two-sample t-test. Significance was set at α = 0.05. All statistical analyses were performed using commercially available software (STATA 13.1; Statacorp LP, College Station, Texas).

## Results

### Patient characteristics

During the study time frame, 105 HMII implants and 34 HVAD implants were performed at our institution. Between the two cohorts, no difference existed with regard to age (*p* = 0.83) and gender with the majority of patients being male (*p* = 0.73, Table 1). The most common indication for LVAD implantation in both groups was destination therapy with 59.1% in the HMII cohort and 52.9% in the HVAD cohort (*p* = 0.89). The second indication for LVAD implantation was bridge to transplantation in 26.7% of the HMII cohort and 35.3% in the HVAD cohort. There was no difference in INTERMACS classification between the two cohorts (*p* = 0.19) with the greatest percentage in both being class 2 with 37.1% in HMII and 41.2% in HVAD.

**Table 1:** Baseline characteristics of patients implanted with the HeartMate II (HMII) and HeartWare (HVAD)

	HMII (*n* = 105)	HVAD (*n* = 34)	*p*-value
Age	56.5 + 13.9	57.2 + 14.6	0.82
Sex male, n (%)	83 (79.1)	27 (81.8)	0.73
Body mass index	29.2 + 6.8	29.5 + 6.2	
LVAD Indication, n (%)			0.89
Bridge to transplantation	28 (26.7)	12 (35.3)	
Destination therapy	62 (59.1)	18 (52.9)	
Bridge to decision	13 (12.4)	1 (2.9)	
Bridge to recovery	2 (1.9)	3 (8.8)	
INTERMACS Classification			0.19
1	12 (14.3)	5 (14.7)	
2	39 (37.1)	14 (41.2)	
3	36 (34.3)	15 (44.1)	
4	14 (13.3)		
5	1 (0.9)		
Heart failure duration, months	2.2 + 1.1	2.3 + 0.8	0.59

In terms of past medical history, there was a low frequency of GI bleeding in both cohorts at less than 4% (*p* = 0.78). For all other past medical history, there was no difference with regards to coronary artery disease (*p* = 0.53), hypertension (*p* = 0.76), atrial fibrillation (*p* = 0.82) and chronic renal insufficiency (*p* = 0.60). The etiology of heart failure was similar between the groups, with ischemic and idiopathic being the two most common causes (*p* = 0.59) (Table 2).

**Table 2:** Pre-implantation past medical history, heart failure, etiology, and laboratory values of patients implanted with the HeartMate II (HMII) and HeartWare (HVAD)

	HMII (*n* = 105)	HVAD (*n* = 34)	*p*-value
Past medical history, *n* (%)			
History of gastrointestinal bleeding	4 (3.9)	1 (2.9)	0.78
Coronary artery disease	49 (46.7)	18 (53.0)	0.53
Diabetes mellitus	48 (45.7)	14 (41.2)	0.65
Smoking	38 (36.2)	22 (66.7)	0.002
Hypertension	55 (52.9)	19 (55.9)	0.76
Atrial fibrillation	44 (41.9)	15 (44.1)	0.82
Cerebral vascular accident	13 (12.4)	3 (8.8)	0.55
Chronic renal insufficiency	37 (35.2)	10 (30.3)	0.60
Heart failure etiology, *n* (%)			0.59
Ischemic	47 (45.2)	12 (55.2)	
Idiopathic	45 (43.3)	10 (34.5)	
Viral	1 (1.0)	–	
Peripartum	4 (3.9)	1 (3.5)	
Alcoholic	1 (1.0)	–	
Myocarditis	1 (1.0)	2 (6.9)	
Chemotherapy Induced cardiomyopathy	2 (1.9)	–	
Vavlular	3 (2.9)	–	
Lab values			
White blood cell (THO/uL)	8.9 + 4.9	7.9 _ 2.5	0.15
Hemoglobin (g/dL)	12.0 + 0.9	10.8 + 0.4	0.23
Platelet (THO/uL)	184.9 + 69.8	191.9 + 83.4	0.66
Prothrombin Time (seconds)	52.6 + 2.5	52.3 + 3.9	0.95

### Intraoperative outcomes

For the HMII cohorts, 21% of the patients were redo-sternotomy compared to 30% in the HVAD (*p* = 0.31). The cardiopulmonary bypass time (85.1 ± 37.7 vs. 81.8 ± 47.9 min, *p* = 0.72) was low and similar in the cohorts. Additionally, there was no difference in blood product transfusion between the two groups (Table 3).

**Table 3:** Intraoperative outcomes and product utilization of patients implanted with the HeartMate II (HMII) and HeartWare (HVAD)

	HMII (*n* = 105)	HVAD (*n* = 34)	*p*-value
Redo sternotomy, *n* (%)	22 (21)	10 (30)	0.31
Cardiopulmonary bypass time, minutes	85.1 + 37.7	81.8 + 47.9	0.72
Product Transfusion, peri-operative (72 h)			
Red blood cells, units	8.2 + 1.0	4.9 + 1.6	0.10
Fresh frozen plasma, units	3.3 + 0.5	2.0 + 0.6	0.19
Platelets, units	1.1 + 0.2	1.2 + 0.5	0.89

### Post-operative outcomes

Within the HVAD cohort, there was a higher requirement for temporary RVAD at 29% compared to 15% in HMII (*p* = 0.02). There was no difference in immediate post-operative day one laboratory values with regards to hemoglobin (9.9 ± 1.5 vs. 9.7 ± 1.8 d/dL, *p* = 0.63) and international normalized ratio (1.4 ± 0.3 vs. 1.3 ± 0.1, *p* = 0.06); however, partial thromboplastin time was greater in the HVAD compared to HMII (34.7 ± 9.3 vs. 40.1 ± 8.4 s, *p* = 0.003).

Prior to return to possible return the operating room, hemodynamics were measured as included in Table 4. Mean arterial pressure was equivalent between the two cohorts (72 ± 11 vs. 68 ± 9 mmHg, *p* = 0.29). As surrogate marker of right ventricular function, central venous pressure was noted to be elevated within the HVAD compared to the HMII cohort (10 ± 3 vs. 13 ± 2 mmHg, *p* = 0.05). Additionally, the cardiac index was reduced in the HVAD cohort compared to HMII (2.5 ± 1.1 vs. 2.2 + 1.3 L/min/m^2^, *p* = 0.04).

**Table 4:** Postoperative outcomes for patients implanted with the HeartMate II (HMII) and HeartWare (HVAD)

	HMII (*n* = 105)	HVAD (*n* = 34)	*p*-value
Temporary RVAD Requirement, *n* (%)	16 (15)	10 (29)	0.02
Time to extubation, days	1.5 (1,5)	2 (1.5)	0.63
ICU length of stay, days	7 (4,14)	8 (6,15)	0.86
Hospital length of stay, days	20 (14,32)	20 (15, 27)	0.59
Post-operative day one lab values			
White blood cells (THO/uL)	15.6 + 6.7	15.5 + 5.7	0.96
Hemoglobin (d/dL)	9.9 + 1.5	9.7 + 1.8	0.63
Platelet (THO/uL)	138.1 + 52.6	147.7 + 57.9	0.39
International normalized ratio	1.4 + 0.3	1.3 + 0.1	0.06
Prothrombin time (seconds)	34.7 + 9.3	40.1 + 8.4	0.003
Lactic acid dehydrogenase (U/L)	499.8 + 228.1	411.6 + 96.1	0.14
Hemodynamics			
Mean Arterial Pressure (mmHg)	72 + 11	68 + 9	0.29
Central Venous Pressure (mmHg)	10 + 3	13 + 2	0.05
Systolic Pulmonary Artery Pressure (mmHg)	28 + 4	26 + 6	0.07
Cardic Index (L/min/m^2^)	2.5 + 1.1	2.2 + 1.3	0.04
Complications- index hospitalization, *n* (%)			
Intra-thoracic bleeding	27 (25.7)	6 (18.2)	0.35
Stroke	4 (3.8)	2 (5.9)	0.65
Re-intubation	20 (19.1)	5 (15.2)	0.6
Drive line infection	2 (3.9)	0 (0.0)	0.05
Gastrointestinal bleeding, *n* (%)			
Index hospitalization	12 (11.5)	2 (5.9)	0.28
3 months	17 (19.5)	3 (11.5)	0.35
12 months	8 (16.33)	0 (0.0)	0.54
24 months	3 (15.0)	0 (0.0)	0.48
36 months	3 (15.0)	0 (0.0)	0.23

Within the index hospitalization, there was a higher but not significantly different incidence of intra-thoracic bleeding requiring operative exploration within the HMII compared to the HVAD cohort (25.7% vs. 18.2%, *p* = 0.35). The rate of GI bleeding within the index hospitalization was similar between the two groups (11.5% vs. 5.9%, *p* = 0.28). Throughout follow-up, there was no difference between GI bleeding at 3, 12, 24, and 36 months (*p* = 0.35, 0.45, 0.48, and 0.23, respectively).

**Fig. 1:**
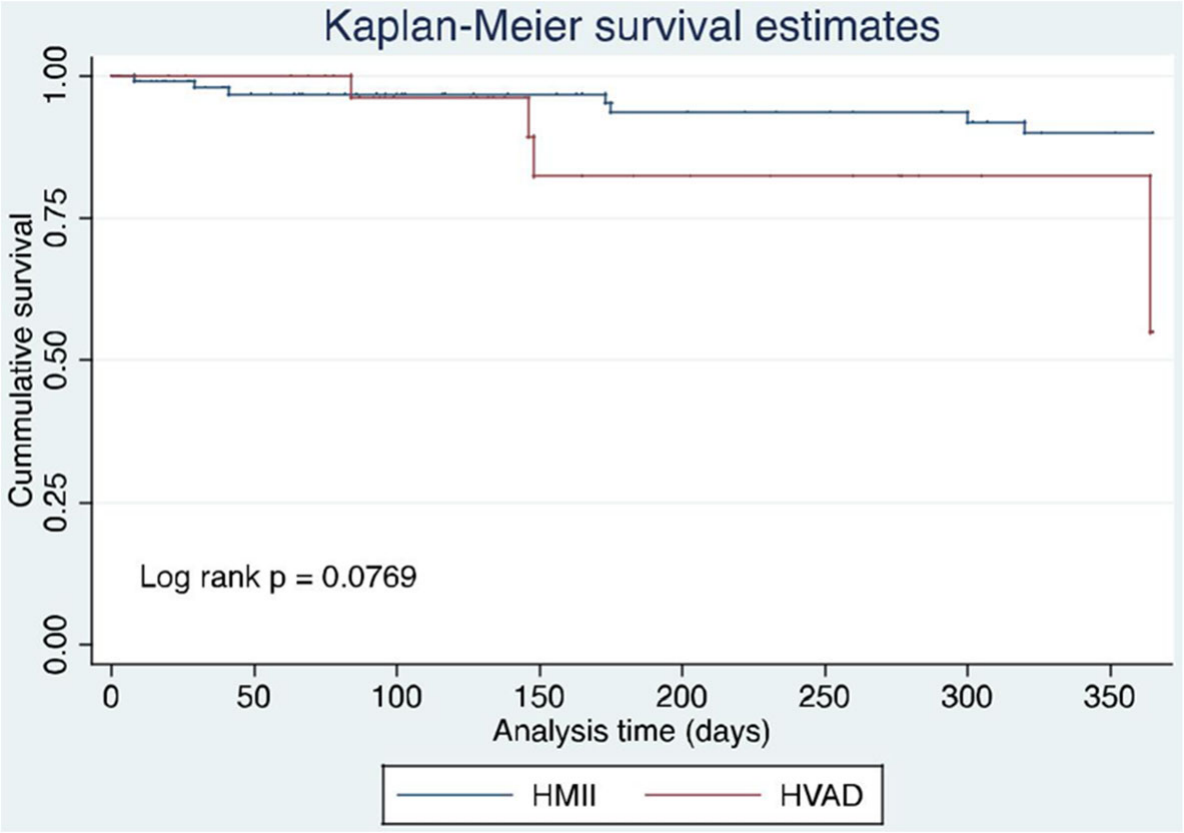
Kaplan Meier survival curve for patients following HMII and HVAD implantation

As shown in Fig. 1, there was no difference in post-VAD implantation survival between the two cohorts (log rank *p* = 0.0769).

## Discussion

Balancing bleeding and thrombotic complications has become a difficult clinical dilemma, made more challenging by the poorly understood difference in risk factors. This study showed that overall, patients had similar outcomes with regards to bleeding, irrespective of whether they received a HMII or HVAD and there was no difference in associated mortality.

Overall, the patient cohorts were similar with regard to pre-operative conditions. The only significant difference between the two cohorts was nearly a one third greater incidence of smoking within the HVAD cohort compared the HMII; however, this pre-operative difference did not translate into a difference in post-operative outcomes. Both groups had a low incidence of prior GI bleeding and with no significant difference in pre-implant coagulation factors. During the operation, and within 30 days post-operatively, there was a low blood product requirement without any difference between the two cohorts.

Post-operatively, a greater percentage of the HVAD cohort required temporary RVAD support. This finding is supported by the reduced right ventricular function within the HVAD cohort immediately prior to RVAD implantation. The CVP was noted to be significantly elevated within the HVAD cohort suggesting right heart strain. The incidence of right ventricular support was higher than that reported in the ADVANCE trial at 2.1% requiring mechanical support and 12.1% on inotropic support [12]. Given that there are no significant differences pre-operatively and intra-operatively between the two groups, it seems as though this difference is likely attributed to small numbers in both cohorts. Overall, both the HMII and HVAD patients had nearly equivalent time to extubation, ICU length of stay, and hospital length of stay.

Furthermore, the incidence of post-operative complications was low between both groups. Examining the implantation technique between the devices, the HMII requires creation and tunneling of subcutaneous pocket while the HVAD has a smaller dissection for the intrapericardial implantation. Despite the differences in technique, no difference existed in overall bleeding events. This similar outcome between the two groups suggests that a meticulous dissection of the pump pocket, as well as hemostasis, is essential to keep bleeding events low for those patients receiving HMII. Additionally, the rate of intra-thoracic bleeding requiring a return to the operating room for a washout was similarly low between the groups. These low rates are further supported by those of the ADVANCE trial with risk of 0.26/patient-year in the HVAD and 0.45/patient-year for the HMII [12].

Furthermore, numerous studies have been published showing that the incidence of GI bleeding after LVAD implantation to vary between 18 and 40% [13–15]. GI bleeding after implantation is classified as upper GI bleeding (proximal to the ligament of Trietz) or lower GI bleeding (distal to the ligament of Trietz). Most common causes are vascular malformations like AVMs and Dieulafoy lesions, which account for 30–40% and 15–20%, respectively [16]. Within our study, the rates of GI bleeding we reported in both cohorts are similar to past studies which reported rates between 11% and 13% [17–19] in the HVAD cohort and 22% in HMII patients [20]. During the index hospitalization only 11.5% of HMII and 5.9% HVAD experienced GI bleeding in our cohort of patients. Throughout follow up there was no difference in the occurrence of a GI bleed between the cohorts out to 36 months of follow-up.

The etiology of the GI bleeding following LVAD implantation is multifactorial given the need for chronic anticoagulation as well as the changes in systemic immunologic and thrombostatic functions [21]. One proposed mechanism of GI bleeding is acquired von Willebrand syndrome due to the fragmentation of high molecular weight multimers of vWF. A study by Meyer et al. [22] demonstrated a reduction in high molecular weight vWF in HVAD patients. This finding suggest that although HVAD shear forces are low due to the contact free design and lower revolutions per minute in the HVAD, the shear force still reaches a sufficient threshold to induce vWF unfolding [23]. As result, that both HMII and HVAD patients develop acquired von Willebrand syndrome, it is anticipated that they would have similar rates of GI bleeding.

For management of an LVAD patient with GI bleeding, a multidisciplinary approach is needed involving possible reversal of anticoagulation, holding of anticoagulants, and possible medical and surgical interventions. Overall, the main goals are to locate the bleeding source and the severity by holding anti-coagulation and resuscitation to maintain stable hemodynamics.

## Conclusions

Overall, balancing bleeding and thrombosis risks continues to be a critical component of LVAD patient management moving forward, especially with the advent of novel anticoagulant agents. Our study further adds to the literature that the rate of bleeding between axial and centrifugal pumps is low without significant difference between the two.

### Limitations

Limitations of this study include its retrospective nature. Due to sample size restrictions, we were unable to perform adjusted analysis. Furthermore, the small sample sized may have limited the study’s ability to detect statistically significant differences in some of the variables compared. The value of transthoracic echocardiogram within the immediate postoperative period is difficult to gain value given the obscure windows, as a result, cardiac index provides a better measurement of function. Additionally, although we did not observe high INR before GI bleeding events, some of the incidences may have been precipitated by high INR in patients.

## Abbreviations

AVMS: Arteriovenous malformations
CF-LVAD: Continuous-flow left ventricular assist devices
GI: Gastrointestinal
HMII: HeartMate II
HVAD: HeartWare
INR: International normalized ratio
INTERMACS: Interagency Registry for Mechanically Assisted Circulatory Support
LVAD: Left ventricular assist device
vWF: Von Willebrand factor (vWF)

## References

[1] Slaughter MS, Rogers JG, Milano CA (2009). Advanced heart failure treated with continuous-flow left ventricular assist device. N Engl J Med.

[2] Genovese EA, Dew MA, Teuteberg JJ (2009). Incidence and patterns of adverse event onset during the first 60 days after ventricular assist device implantation. Ann Thorac Surg.

[3] Pagani FD, Miller LW, Russell SD (2009). Extended mechanical circulatory support with a continuous-flow rotary left ventricular assist device. J Am Coll Cardiol.

[4] Uriel N, Pak S-W, Jorde UP (2010). Acquired von Willebrand syndrome after continuous-flow mechanical device support contributes to a high prevalence of bleeding during long-term support and at the time of transplantation. J Am Coll Cardiol.

[5] Pal JD, Piacentino V, Cuevas AD (2009). Impact of left ventricular assist device bridging on posttransplant outcomes. Ann Thorac Surg.

[6] Jessup ML, Goldstein D, Ascheim DD, et al. 5 risk for bleeding after MCSD implant: an analysis of 2358 patients in INTERMACS. J Hear Lung Transplant. 2011;30(4) 10.1016/j.healun.2011.01.012.

[7] Stern DR, Kazam J, Edwards P (2010). Increased incidence of gastrointestinal bleeding following implantation of the HeartMate II LVAD. J Card Surg.

[8] Stulak JM, Mehta V, Schirger JA, et al. Temporal Differences in Causes of Mortality After Left Ventricular Assist Device Implantation. Ann Thorac Surg. 2015;99(6):1969-74. 10.1016/j.athoracsur.2015.01.036. 10.1016/j.athoracsur.2015.01.036 25865761

[9] Geisen U, Heilmann C, Beyersdorf F (2008). Non-surgical bleeding in patients with ventricular assist devices could be explained by acquired von Willebrand disease. Eur J Cardiothorac Surg.

[10] Crow S, John R, Boyle A (2009). Gastrointestinal bleeding rates in recipients of nonpulsatile and pulsatile left ventricular assist devices. J Thorac Cardiovasc Surg.

[11] Suarez J, Patel CB, Felker GM, Becker R, Hernandez AF, Rogers JG (2011). Mechanisms of bleeding and approach to patients with axial-flow left ventricular assist devices. Circ Heart Fail.

[12] Aaronson KD, Slaughter MS, Miller LW, et al. Use of an intrapericardial, continuous-flow, centrifugal pump in patients awaiting heart transplantation. Circulation. 2012;125(25):3191-200. 10.1161/CIRCULATIONAHA.111.058412. 10.1161/CIRCULATIONAHA.111.058412 22619284

[13] John R, Kamdar F, Eckman P (2011). Lessons learned from experience with over 100 consecutive HeartMate II left ventricular assist devices. Ann Thorac Surg.

[14] Morgan JA, Paone G, Nemeh HW (2012). Gastrointestinal bleeding with the HeartMate II left ventricular assist device. J Heart Lung Transplant.

[15] Aggarwal A, Pant R, Kumar S (2012). Incidence and management of gastrointestinal bleeding with continuous flow assist devices. Ann Thorac Surg.

[16] Demirozu ZT, Radovancevic R, Hochman LF (2011). Arteriovenous malformation and gastrointestinal bleeding in patients with the HeartMate II left ventricular assist device. J Heart Lung Transplant.

[17] Wieselthaler GM, Gerry O, Jansz P, Khaghani A, Strueber M (2010). Initial clinical experience with a novel left ventricular assist device with a magnetically levitated rotor in a multi-institutional trial. J Heart Lung Transplant.

[18] Miller LW, Pagani FD, Russell SD, et al. Use of a Continuous-Flow Device in Patients Awaiting Heart Transplantation. N Engl J Med. 2007;357(9):885-96. 10.1056/NEJMoa067758. 10.1056/NEJMoa067758 17761592

[19] Popov AF, Hosseini MT, Zych B (2012). Clinical experience with HeartWare left ventricular assist device in patients with end-stage heart failure. Ann Thorac Surg.

[20] Demirozu ZT, Radovancevic R, Hochman LF, et al. Arteriovenous malformation and gastrointestinal bleeding in patients with the HeartMate II left ventricular assist device. J Hear Lung Transplant. 2011;30(8):849-53. 10.1016/j.healun.2011.03.008. 10.1016/j.healun.2011.03.008 21530318

[21] John R, Lee S (2009). The biological basis of thrombosis and bleeding in patients with ventricular assist devices. J Cardiovasc Transl Res.

[22] Meyer AL, Malehsa D, Budde U, Bara C, Haverich A, Strueber M (2014). Acquired von Willebrand syndrome in patients with a centrifugal or axial continuous flow left ventricular assist device. JACC Heart Fail..

[23] Crow SS, Joyce DD (2014). Are centrifugal ventricular assist devices the answer to reducing post-implantation gastrointestinal bleeding?. JACC Heart Fail.

